# Effect of the COVID-19 Pandemic on Healthy Components of Diet and Factors Associated with Unfavorable Changes among University Students in France

**DOI:** 10.3390/nu14183862

**Published:** 2022-09-18

**Authors:** Lise Miller, Pierre Déchelotte, Joel Ladner, Marie-Pierre Tavolacci

**Affiliations:** 1 Department of Epidemiology and Health Promotion, CHU Rouen, F 76000 Rouen, France; 2 Department of Nutrition, CHU Rouen, U 1073, Normandie University, UNIROUEN, F 76000 Rouen, France; 3 Department of Epidemiology and Health Promotion, CHU Rouen, U 1073, Normandie University, UNIROUEN, F 76000 Rouen, France; 4 Clinical Investigation Center 1404, CHU Rouen, U 1073, Normandie University, UNIROUEN, F 76000 Rouen, France

**Keywords:** COVID-19, university students, diet quality, PNNS-GS2

## Abstract

Introduction: The COVID-19 pandemic and the lockdowns have affected many aspects of university students’ daily lives, including their dietary habits. This study aimed to evaluate the change of diet quality of university students before and during the COVID-19 period, and the factors associated with unfavorable changes in diet quality. Methods: An online cross-sectional study was performed in May 2021 among Rouen (France) university students. Socio-demographic characteristics, body mass index, depression, academic stress, risk of eating disorders and food security were collected. The French “Programme National Nutrition Santé-Guidelines Score 2” (PNNS-GS2) was used to access diet quality. Results: A total of 3508 students were included, 74.4% were female, the mean age was 20.7 (SD = 2.3), and 7.0% were in a situation of food insecurity. The PNNS-GS2 score decreased between the pre- and the COVID-19 pandemic period for 33.1% of university students. The associated factors with the decrease in the PNNS-GS2 score were food insecurity, financial insecurity, not living with parents, depression, academic stress, eating disorders, being in the two first years of study and having been infected by COVID-19. Conclusions: Diets with healthy components decreased for one-third of university students since the COVID-19 pandemic, and this was shown to be associated with food insecurity, poor mental health and eating disorder. This study provides important information to help public health authorities and universities give better support to student health feeding programs during pandemics and lockdowns.

## 1. Introduction

The year 2020 was highly impacted by the coronavirus disease 19 (COVID-19) outbreak. It was declared by the World Health Organization (WHO) as a public health emergency in January 2020 and categorized as a world pandemic in March of 2020 [1]. In order to reduce the virus transmission, countries worldwide had to take extreme measures. In France, the first lockdown was implemented on 17 March 2020 [2]. This measure was partially lifted on 11 May 2020 [3]. A second lockdown occurred in November 2020 and was lifted in mid-December 2020, and a third occurred between 3 April and 3 May 2021 [4] with lighter restrictive measures such as curfews and various restrictions on going out in public. During the lockdowns, people had to stay at home and only “essential” activities such as medical activities and food shopping were permitted. Universities immediately closed and left students with many uncertainties and much stress pertaining to how they would successfully complete the academic year [5]. Online teaching developed quickly, and examination methods had to be redesigned. Student were particularly impacted by the crisis and the restrictive measures of the lockdowns [6]. Some students had to face a loss of their main source of income and suffer financial insecurity leading to food insecurity [7,8]. Poorer dietary outcomes were found in university students with food insecurity compared with food-secure students in the few studies performed before the COVID-19 pandemic [9]. Student mental health was poorer during this period due to the prevalence of higher stress levels, depression symptoms and eating disorders [10,11,12].

This period of social restrictions designed to limit the transmission of the virus led to changes in lifestyle for most of the general population, especially changes in dietary habits [13,14]. Studies showed that people tend to have more unhealthy food consumption and meal patterns during lockdowns [15]. Sales in fresh food, fresh fruits, vegetables and fish decreased during lockdowns [16], whereas the consumption of comfort food and snacks increased compared to before the lockdown [17]. Overall, student diet quality was poorer with less fruit and vegetable consumption and more snacking [18,19]. It appears important to continue to evaluate food behavior in university students during the COVID-19 pandemic period one year after the first lockdown. The aim of this study was to assess the components relating to a healthy student diet before and during the COVID-19 pandemic period in May 2021 and to identify associated factors leading to unfavorable changes in diet quality.

## 2. Materials and Methods

### 2.1. Study Design and Participants

A cross-sectional study was conducted at University of Rouen (Rouen, Normandy region, France) with an anonymous online questionnaire sent by email in May 2021. Participants included were students aged 18 or more, and in the University of Rouen. The Rouen University Hospital’s Institutional Review Board, without mandatory informed consent (E2020-22), approved the observational study.

### 2.2. Data Collection

#### 2.2.1. Socio Demographics Characteristics

These characteristics included gender, age, curriculum (categorized in four curricula: Law and Economics, Healthcare, Literature and Humanities, and Sciences) and academic year of study (four categories: year 1, year 2, year 3, and year 4 and more). Students provided data regarding their living situation: with their parents or not. Students were asked whether they had sufficient financial resources during the COVID-19 lockdown. Students were also asked if they had been infected by COVID-19. Students’ weight and height were also collected in order to calculate the body mass index (BMI) using the standard formula (BMI weight [kg]/height [m^2^]) and were classified as: underweight (BMI < 18.5), normal weight (BMI between 18.5 and 24.9), overweight (BMI between 25.0 and 29.9) and obesity (upper 30.0)

#### 2.2.2. Eating Habits

The French dietary guideline score “PNNS-GS2” (Programme National Nutrition Santé, Guidelines Score 2) was developed by Chaltiel et al. [20]. Cut-offs and associated scorings were defined with the support of nutrition experts who were involved in the development of the guidelines of the 2017 French dietary recommendations [21]. PNNS-GS2 was used to assess the healthier components: fruits and vegetables, nuts, legumes, cereals, dairy and fish. For example, one question was the following: “*On average, how many portions (80 g) of fruit and vegetables do you eat per DAY” during the pre-COVID-19 period and the last month*.” For each component, 1.0 to 2.0 points were accorded if the student reached the guideline and 0.0 to 0.5 points if the guideline was not reached (Table 1). The total score was standardized and weighted according to the level of evidence of the association between food groups and health: a weight of 3 for fruits and vegetables, 2 for cereals and fish and 1 for nuts, legumes and dairy products were calculated to according this formula: *Score PNNS GS*2 = Σ(*componentii* × *weighti*max (*abs*(*componenti*))) [20]. The PNNS-GS2 score ranked from 0 to 14. A higher score indicates a better diet, closer to the national guidelines. Data were retrospectively collected for the two periods: the pre-COVID-19 period and the COVID-19 period (May 2021). Two groups of university students were identified according to the change of their PNNS-GS2 score: students with a decrease, and students with an increase or no change between the two studied periods. Food security was established using the validated six-item Food Security Survey Module (FSSM): a score of 0–1 indicated food security; 2–4, low food security; and 5–6, food insecurity [22]. Eating disorders were assessed by the French version of the validated five-item SCOFF (Sick, Control, One stone, Fat, Food) questionnaire to evaluate if they presented an eating disorder or not [23].

### 2.3. Mental Health

Depression was measured using the validated CESD-8 (Center for Epidemiologic Studies-Depression) scale [24]. A score was established with a range from 0 to 24; higher scores indicated a higher frequency of depressive behaviors. Academic stress was assessed with four items: “increased academic workload”, “stress with changes in teaching methods”, “concern about not being able to validate the academic year” and “difficulty in keeping up with e-learning courses”, using a Likert scale (0 totally disagree to 4 totally agree). A score from 0 to 16 was calculated.

### 2.4. Statistical Analysis

Qualitative variables were reported as percentages and continuous variables as mean with standard deviation (SD). Comparisons between before and during the COVID-19 period (May 2021) were obtained with a McNemar test for paired qualitative data. Concerning the PNNS-GS2 score, the comparison was conducted with a Student *t*-test for paired data. Comparisons for each variable in terms of changes in PNNS-GS2 score were obtained with a Chi2 test for independent qualitative data. Bonferroni’s post-hoc correction was performed for univariate analysis, with a *p <* 0.004 as significant.

To identify factors associated with a decrease in the PNNS-GS2 score, a multivariate logistic regression was fitted. Variables with a *p <* 0.20 in univariate analysis were included in the multivariate model. Adjusted odds ratios (AOR) and 95% confidence intervals (95% CI) were presented. All statistics were made using R.

## 3. Results

A total of 3508 students were included (response rate: 11.7%) with a mean age of 20.7 years (SD = 2.0 years) and 74.4% were women. The students were mostly in the first year of study (36.3%) and in a healthcare curriculum (31.4%). During the COVID-19 pandemic, 49.0% of the students did not live with their parents. Mild and food insecurity concerned 11.3% and 7.0% of the university students, respectively. The risk of eating disorder was detected by the SCOFF test among 46.6% of the university students (Table 2).

**Figure 1:**
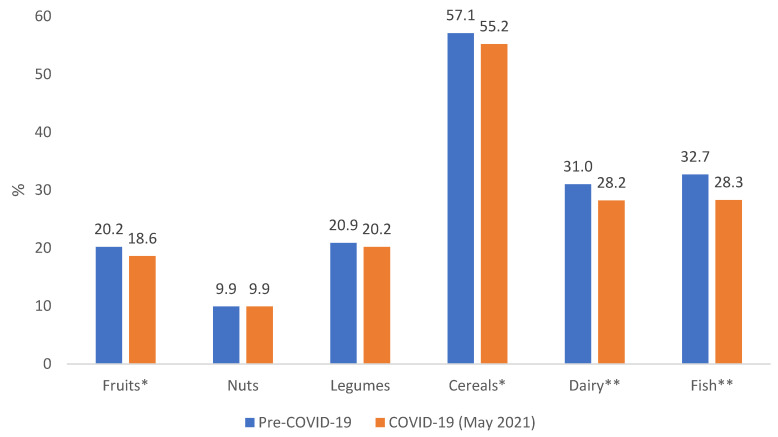
Compliance with the national dietary guideline components in the pre-COVID-19 period and the COVID-19 period (May 2021) according to the components among university students (*n =* 3508). * *p <* 0.05,** *p <* 0.001.

**Figure 2:**
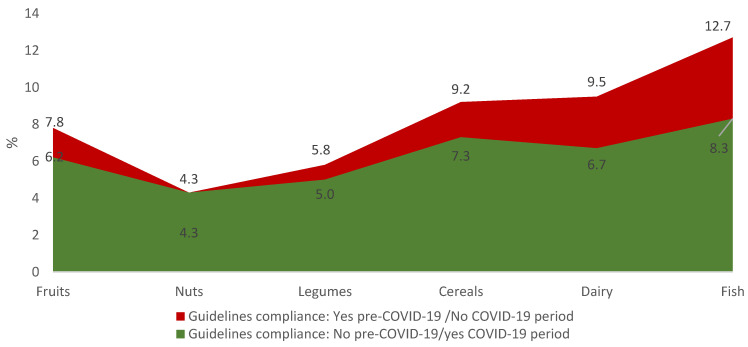
Improving or worsening of the compliance with the national dietary guidelines between the pre-COVID-19 and the COVID-19 period (May 2021) according to the components among university students (*n =* 3508).

The PNNS-GS2 score was 5.0 (SD = 2.3) in the pre-COVID-19 period and 4.7 (SD = 2.3) in the COVID-19 period (*p <* 0.0001), for women it was 4.9 (SD = 2.3) and 4.6 (SD = 2.3) (*p <* 0.0001), and for men it was 5.3 (SD = 2.3) and 4.9 (SD = 2.5) (*p <* 0.0001). The PNNS-GS2 score decreased for 33.1% of the university students between these two periods. Cereals were the only food which had more than 50% of compliance with the dietary recommendations. Significant decreases were found between the pre-COVID-19 and the COVID-19 period for the consumption of fruits (*p <* 0.01), cereals (*p <* 0.01), fish (*p <* 0.0001 and dairy (*p <* 0.0001) (Figure 1). Figure 2 specifies the improvement and the worsening of the compliance with the national dietary guidelines between the pre- COVID-19 and the COVID-19 period (May 2021) according to the components.

Factors significantly associated with the decrease in the PNNS-GS2 score were the following: being in science curricula, not living with parents, having been infected by COVID-19, not having enough financial resources, being in a food insecurity situation, CESD-8 scores, academic stress scores and having an eating disorder (Table 3).

## 4. Discussion

This study highlights the change in healthy components of the diet of university students according to the French dietary guideline one year after the beginning of the COVID-19 pandemic. Even before the COVID-19 pandemic, students did not meet the recommendations of the French guidelines. These findings are congruent with the 2019 report of “Santé Publique France” that showed that French adults are not in agreement with the guidelines [25]. In order to improve student diet quality, university restaurants must serve balanced meals with healthy food options for a period of several years in order to help students to eat healthier [26]. More than one year after the beginning of the pandemic, unfavorable changes in diet quality were observed among students and according to our results, it is likely that the crisis aggravated this failure to meet the recommendations. Factors associated with those unfavorable changes included: science curricula, not living with parents, having been infected by COVID-19, financial resources, food insecurity, depression, academic stress and eating disorders. A systematic review of studies performed in November 2020 showed that the diet quality of college students was compromised during the pandemic due to the decrease in the intake of healthy food [8]. The principal goal of the lockdown was to reduce the transmission of the virus by limiting social interactions, but a lack of social support can negatively affect student mental health [27]. In our study, depression was associated with a decrease in the PNNS-GS2. A systemic review performed before the COVID-19 pandemic already showed an association between a high-quality diet and a lower risk of depression [28], and this was confirmed during the COVID-19 crisis [29,30]. During that crisis, the changes in teaching modalities created much stress for students (45–48). As in our study, studies have shown that academic stress leads to unhealthier eating behaviors [31]. The French government implemented during the crisis “*SantéPsy*”, a program that allows students to consult a psychologist for free [32]. This measure needs to be further investigated to understand to what extent it helped students during the crisis. Accordingly, it appears important to support student mental health issues and behaviors towards food. For lockdown periods, the development of prevention tools that are remotely accessible seems necessary. Holland et al. showed that school-based telehealth could offer young people a safe therapeutic practice [33].

Galanakis and al. highlighted the rise of food insecurity around the world due to the COVID-19 crisis [34]. We found that the food insecurity is associated with a PNNS-GS2 decrease [35]. In France, six out of ten students had to stop or reduce their job activities and they were particularly affected by food insecurity [36,37,38]. To prevent food insecurity, the French government established the “repas à 1 euro” (1-euro meal) in the university restaurant, first for scholarship holders then for all students [39]. An alternative explored by Buscail and al. before the COVID-19 pandemic is the implementation of fruit and vegetable vouchers to relieve food insecurity and promote healthy food [40]. Students with a risk of eating disorders have decreased their diet quality, which can be explained by an increase in eating disorders since the beginning of the COVID-19 pandemic [10].

Further research needs to keep investigating the food behavior of university students. Longitudinal studies could explore the long-term effect of the pandemic on student diet and the impact of solutions implemented by the French government. At the same time, university-wide prevention programs could be a good way to encourage students to adopt healthier food behaviors and prevent mental health issues. Continuing to develop healthy options in university restaurants and preventing food insecurity and mental health issues must be priorities among French universities.

This study presents several limitations. It was a convenience sample and could limit extrapolation to the results. The percentage of women and healthcare students is higher than in the population of the University of Rouen. Data on reported food consumption may lead to misreporting, most often underreporting [41]. In addition, the dietary questionnaire asked about consumption more than one year ago, which may lead to memorization bias. Nevertheless, this was not a 24-h dietary recall but focused on usual consumption patterns that do not require as much accuracy. The design was cross-sectional and did not allow causal interpretation between risk factors and unfavorable changes in quality. The main strength is its statistical power due to the large sample size.

## 5. Conclusions

The student population was particularly impacted by the COVID-19 crisis, with an unfavorable change in diet quality for one-third of the university students associated with food insecurity, poor mental health and eating disorders. Universities have a role to play to protect the health of their students, especially in periods of crisis. Further research could help develop innovative prevention programs that would seek to promote healthy food and mental health at all times.

## Figures and Tables

**Table 1:** Programme National Nutrition Santé—guidelines score 2 (PNNS-GS2): components and scoring.

	Servings	Score
Fruits and vegetables (weight 3) 5 servings/day	0–3.5 3.5–5 5–7.5 >7.5	0 0.5 1 2
Nuts (weight 1) 1 handful/day	0 0–0.5 0.5–1.5 >1.5	0 0.5 1 0
Legumes (weight 1) 2 servings/week	0 0–2 >2	0 0.5 1
Cereals (weight 2) Every day	0 0–1 1–2 >2	0 0.5 1 1.5
Dairy (weight 1) 2 servings/day	0–0.5 0.5–1.5 1.5–2.5 >2.5	0 0.5 1 0
Fish (weight 2) 2 servings/week	0–1.5 1.5–2.5 2.5–3.5 >3.5	0 1 0.5 0

PNNS-GS2 (Programme National Nutrition Santé, Guidelines Score 2); National guidelines: Score ≥ 1 Adapted from Chaltiel D et al.

**Table 2:** Characteristics of the university students according to the change of the PNNS-GS2 (*n =* 3508).

	Decrease in PNNS-GS2 *n =* 1163	No Change or Increase in PNNS-GS2 *n =* 2345	Total *n =* 3508	*p*
Women (%)	77.0	73.0	74.4	0.01
Age mean (SD)	20.7 (2.3)	20.8 (2.3)	20.7 (2.3)	0.40
Curriculum (%)				0.07
Healthcare	28.7	32.8	31.4	
Law and Economics	18.1	17.4	17.6	
Literature and Humanities	26.5	26.1	26.2	
Sciences	26.7	23.7	24.7	
Academic year of study (%)				0.007
1	38.8	35.1	36.3	
2	26.4	26.0	26.1	
3	17.9	17.2	17.4	
4 and more	16.9	21.7	20.2	
Not living with parents (%)	61.6	42.8	49.0	<0.0001
Insufficient financial resources (%)	31.0	12.9	18.9	<0.0001
Have been infected by COVID 19 (%)	21.6	17.0	18.5	<0.0001
Body Mass Index (%)				0.39
Underweight	10.7	10.7	10.7	
Normal weight	69.9	70.7	70.4	
Overweight	13.2	13.7	13.5	
Obesity	6.2	4.9	5.4	
Food security (%)				<0.0001
Security	66.2	89.4	81.7	
Mild insecurity	19.7	7.2	11.3	
Insecurity	14.1	3.4	7.0	
Eating disorders (%)	58.6	40.6	46.6	<0.0001
Depression/24 mean (SD)	12.9 (5.8)	10.2 (5.8)	11.1 (5.9)	<0.0001
Academic stress/16 mean (SD)	12.0 (3.3)	10.6 (3.8)	11.0 (3.7)	<0.0001

PNNS-GS2: Programme National Nutrition Santé, Guidelines Score 2.

**Table 3:** Factors associated with the decrease in the PNNS-GS2; logistic regression (*n =* 3508).

	AOR 95% CI	*p*
Women	1.13 (0.93–1.37)	0.09
Curriculum		
Healthcare	1.04 (0.83–1.31)	0.91
Law and Economics	Ref	
Literature and Humanities	0.99 (0.79–1.25)	0.77
Sciences	1.28 (1.00–1.62)	0.05
Academic year of study		
1	1.61 (1.29–2.01)	<0.0001
2	1.34 (1.05–1.70)	0.01
3	1.32 (1.02–1.70)	0.03
4 and more	Ref	
Not living with parents	1.86 (1.59–2.2)	<0.0001
Insufficient financial resources	1.47 (1.18–1.81)	<0.0001
Have been infected by COVID 19	1.28 (1.06–1.56)	0.01
Food security		
Security	Ref	
Mild insecurity	2.26 (1.78–2.88)	<0.0001
Insecurity	2.71 (1.97–3.74)	<0.0001
Eating disorders	1.38 (1.17–1.63)	<0.0001
Depression	1.03 (1.02–1.05)	<0.0001
Academic stress	1.05 (1.02–1.07)	<0.0001

AOR: Adjusted Odds Ratio; CI: Confidence Interval.

## Data Availability

Data are available on request.

## References

[1] WHO Director-General’s Opening Remarks at the Media Briefing on COVID-19—15 May 2020. https://www.who.int/director-general/speeches/detail/who-director-general-s-opening-remarks-at-the-media-briefing-on-covid-19---15-may-2020.

[2] Décret n° 2020-260 du 16 Mars 2020 Portant Réglementation des Déplacements Dans le Cadre de la Lutte Contre la Propagation du Virus COVID-19. https://www.legifrance.gouv.fr/loda/id/JORFTEXT000041728476/.

[3] Décret n° 2020-545 du 11 Mai 2020 Prescrivant les Mesures Générales Nécessaires Pour Faire Face à L’épidémie de COVID-19 dans le Cadre de L’état D’urgence Sanitaire. https://www.legifrance.gouv.fr/loda/id/JORFTEXT000041858681/.

[4] Légifrance Décret n° 2020-1331 du 2 Novembre 2020 Modifiant le Décret n° 2020-1310 du 29 Octobre 2020 Prescrivant les Mesures Générales Nécessaires Pour Faire Face à L’épidémie de COVID-19 Dans le Cadre de L’état D’urgence Sanitaire. https://www.legifrance.gouv.fr/jorf/id/JORFTEXT000042486870.

[5] Butt S., Mahmood A., Saleem S. (2022). The role of institutional factors and cognitive absorption on students’ satisfaction and performance in online learning during COVID 19. PLoS ONE.

[6] Arsandaux J., Montagni I., Macalli M., Texier N., Pouriel M., Germain R., Mebarki A., Kinouani S., Tournier M., Schuck S. (2021). Mental health condition of college students compared to non-students during COVID-19 lockdown: The CONFINS study. BMJ Open.

[7] Bruening M., Brennhofer S., Van Woerden I., Todd M., Laska M. (2016). Factors related to the high rates of food insecurity among diverse, urban college freshmen. J. Acad. Nutr. Diet..

[8] Jehi T., Khan R., Halawani R., Dos Santos H. (2022). Effect of COVID-19 Outbreak on the Diet, Body Weight, and Food Security Status of Students of Higher Education: A Systematic Review. Br. J. Nutr..

[9] Shi Y., Davies A., Allman-Farinelli M. (2021). The Association Between Food Insecurity and Dietary Outcomes in University Students: A Systematic Review. J. Acad. Nutr. Diet..

[10] Tavolacci M.P., Ladner J., Déchelotte P. (2021). Sharp Increase in Eating Disorders among University Students since the COVID-19 Pandemic. Nutrients.

[11] Huckins J.F., Dasilva A.W., Wang W., Hedlund E., Rogers C., Nepal S.K., Wu J., Obuchi M., Murphy E.I., Meyer M.L. (2020). Mental Health and Behavior of College Students During the Early Phases of the COVID-19 Pandemic: Longitudinal Smartphone and Ecological Momentary Assessment Study. J. Med. Internet Res..

[12] Tavolacci M.P., Ladner J., Dechelotte P. (2021). COVID-19 Pandemic and Eating Disorders among University Students. Nutrients.

[13] Giacalone D., Frøst M.B., Rodríguez-Pérez C. (2020). Reported Changes in Dietary Habits During the COVID-19 Lockdown in the Danish Population: The Danish COVIDiet Study. Front. Nutr..

[14] Janssen M., Chang B.P.I., Hristov H., Pravst I., Profeta A., Millard J. (2021). Changes in Food Consumption During the COVID-19 Pandemic: Analysis of Consumer Survey Data From 18 the First Lockdown Period in Denmark, Germany, and Slovenia. Front. Nutr..

[15] Ammar A., Brach M., Trabelsi K., Chtourou H., Boukhris O., Masmoudi L., Bouaziz B., Bentlage E., How D., Ahmed M. (2020). Effects of COVID-19 Home Confinement on Eating Behaviour and Physical Activity: Results of the ECLB-COVID19 International Online Survey. Nutrients.

[16] Bracale R., Vaccaro C.M. (2020). Changes in food choice following restrictive measures due to Covid-19. Nutr. Metab. Cardiovasc. Dis..

[17] Deschasaux-Tanguy M., Druesne-Pecollo N., Esseddik Y., De Edelenyi F.S., Allès B., Andreeva V.A., Baudry J., Charreire H., Deschamps V., Egnell M. (2021). Diet and physical activity during the coronavirus disease 2019 (COVID-19) lockdown (March–May 2020): Results from the French NutriNet-Santé cohort study. Am. J. Clin. Nutr..

[18] Bertrand L., Shaw K.A., Ko J., Deprez D., Chilibeck P.D., Zello G.A. (2021). The impact of the coronavirus disease 2019 (COVID-19) pandemic on university students’ dietary intake, physical activity, and sedentary behaviour. Appl. Physiol. Nutr. Metab..

[19] Palmer K., Bschaden A., Stroebele-Benschop N. (2021). Changes in lifestyle, diet, and body weight during the first COVID 19 ‘lockdown’ in a student sample. Appetite.

[20] Chaltiel D., Adjibade M., Deschamps V., Touvier M., Hercberg S., Julia C., Kesse-Guyot E. (2019). Programme National Nutrition Santé—Guidelines score 2 (PNNS-GS2): Development and validation of a diet quality score reflecting the 2017 French dietary guidelines. Br. J. Nutr..

[21] High Council for Public Health (2017). Statement Related to the Revision of the 2017–2021 French Nutrition and Health Programme’s Dietary Guidelines for Adults. https://www.hcsp.fr/Explore.cgi/AvisRapportsDomaine?clefr=653.

[22] Blumberg S.J., Bialostosky K., Hamilton W.L., Briefel R.R. (1999). The effectiveness of a short form of the Household Food Security Scale. Am. J. Public Health.

[23] Tavolacci M.P., Gillibert A., Zhu Soubise A., Grigioni S., Déchelotte P. (2019). Screening four broad categories of eating disorders: Suitability of a clinical algorithm adapted from the SCOFF questionnaire. BMC Psychiatry.

[24] Van de Velde S., Levecque K., Bracke P. (2009). Measurement equivalence of the CES-D 8 in the general population in Belgium: A gender perspective. Arch. Public Health.

[25] Santé Publique France Adéquation aux Nouvelles Recommandations Alimentaires des Adultes Âgés de 18 à 54 ans Vivant en France: Étude Esteban 2014–2016. https://www.santepubliquefrance.fr/determinants-de-sante/nutrition-et-activite-physique/adequation-aux-nouvelles-recommandations-alimentaires-des-adultes-ages-de-18-a-54-ans-vivant-en-france-etude-esteban-2014-2016.-volet-nutrition.

[26] Pavaut B., Vieux F., Darmon N. (2011). Le Resto’U: Une aide pour respecter le PNNS?. Cah. Nutr. Diététique.

[27] Elmer T., Mepham K., Stadtfeld C. (2020). Students under lockdown: Comparisons of students’ social networks and mental health before and during the COVID-19 crisis in Switzerland. Capraro V, éditeur. PLoS ONE.

[28] Molendijk M., Molero P., Sánchez-Pedreño F.O., Van der Does W., Martínez-González M.A. (2018). Diet quality and depression risk: A systematic review and dose-response meta-analysis of prospective studies. J. Affect. Disord..

[29] Pham K.M., Pham L.V., Phan D.T., Tran T.V., Nguyen H.C., Nguyen M.H., Nguyen H.C., Ha T.H., Dao H.K., Nguyen P.B. (2020). Healthy Dietary Intake Behavior Potentially Modifies the Negative Effect of COVID-19 Lockdown on Depression: A Hospital and Health Center Survey. Front. Nutr..

[30] LaCaille L.J., Hooker S.A., Marshall E., LaCaille R.A., Owens R. (2021). Change in Perceived Stress and Health Behaviors of Emerging Adults in the Midst of the COVID-19 Pandemic. Ann. Behav. Med..

[31] Ramón-Arbués E., Gea-Caballero V., Granada-López J.M., Juárez-Vela R., Pellicer-García B., Antón-Solanas I. (2020). The Prevalence of Depression, Anxiety and Stress and Their Associated Factors in College Students. Int. J. Env. Res. Public Health.

[32] Santé Psy Étudiants, un Dispositif de Soutien Psychologique aux Étudiants. https://www.enseignementsup-recherche.gouv.fr/fr/sante-psy-etudiants-un-dispositif-de-soutien-psychologique-aux-etudiants-49199.

[33] Holland M., Hawks J., Morelli L.C., Khan Z. (2021). Risk Assessment and Crisis Intervention for Youth in a Time of Telehealth. Contemp. Sch. Psychol..

[34] Galanakis C.M. (2020). The Food Systems in the Era of the Coronavirus (COVID-19) Pandemic Crisis. Foods.

[35] El Zein A., Shelnutt K.P., Colby S., Vilaro M.J., Zhou W., Greene G., Olfert M.D., Riggsbee K., Morrell J.S., Mathews A.E. (2019). Prevalence and correlates of food insecurity among U.S. college students: A multi-institutional study. BMC Public Health.

[36] Mialki K., House L.A., Mathews A.E., Shelnutt K.P. (2021). COVID-19 and College Students: Food Security Status before and after the Onset of a Pandemic. Nutrients.

[37] Niles M.T., Bertmann F., Belarmino E.H., Wentworth T., Biehl E., Neff R. (2020). The Early Food Insecurity Impacts of COVID-19. Nutrients.

[38] Arnault E. (2021). COVID-19: Les étudiants face à la crise. Presse Médicale.

[39] Un Repas à un Euro Pour Tous les Étudiants Dans Tous les CROUS. https://www.enseignementsup-recherche.gouv.fr/fr/un-repas-un-euro-pour-tous-les-etudiants-dans-tous-les-crous-46897.

[40] Buscail C., Gendreau J., Daval P., Lombrail P., Hercberg S., Latino-Martel P., Julia C. (2019). Impact of fruits and vegetables vouchers on food insecurity in disadvantaged families from a Paris suburb. BMC Nutr..

[41] Castro-Quezada I., Ruano-Rodríguez C., Ribas-Barba L., Serra-Majem L. (2015). Misreporting in nutritional surveys: Methodological implications. Nutr. Hosp..

